# Structural design of the minute clypeasteroid echinoid *Echinocyamus pusillus*

**DOI:** 10.1098/rsos.171323

**Published:** 2018-05-09

**Authors:** Tobias B. Grun, James H. Nebelsick

**Affiliations:** Department of Geosciences, University of Tübingen, Hölderlinstraße 12, 72074 Tübingen, Germany

**Keywords:** echinoid skeleton, multi-element shell, structural hierarchy, plate joints, internal supports, stereom architecture

## Abstract

The clypeasteroid echinoid skeleton is a multi-plated, light-weight shell construction produced by biomineralization processes. In shell constructions, joints between individual elements are considered as weak points, yet these echinoid skeletons show an extensive preservation potential in both Recent and fossil environments. The remarkable strength of the test is achieved by skeletal reinforcement structures and their constructional layouts. Micro-computed tomography and scanning electron microscopy are used for microstructural and volumetric analyses of the echinoid's skeleton. It is shown that strengthening mechanisms act on different hierarchical levels from the overall shape of the skeleton to skeletal interlocking. The tight-fitting and interlocking plate joints lead to a shell considered to behave as a monolithic structure. The plate's architecture features distinct regions interpreted as a significant load-transferring system. The internal support system follows the segmentation of the remaining skeleton, where sutural layout and stereom distribution are designed for effective load transfer. The structural analysis of the multi-plated, yet monolithic skeleton of *Echinocyamus pusillus* reveals new aspects of the micro-morphology and its structural relevance for the load-bearing behaviour. The analysed structural principles allow *E. pusillus* to be considered as a role model for the development of multi-element, light-weight shell constructions.

## 1. Introduction

Organisms have developed various strategies to reinforce their skeletons during evolution [1]. Some of these skeletons are of high interest for technical solutions as their underlying constructional principles can be transferred to architecture and engineering disciplines. Hereby, existing solutions for technical problems can be improved or new strategies for structural optimization can be developed [2–4]. Dome shaped objects [5,6], as well as the suturing and interlocking mechanisms between skeletal elements in both vertebrate and invertebrate skeletons have been analysed with respect to their mechanical design and function [7,8].

In architecture and engineering sciences, structural configurations are obviously of major interest with domes, for example, demonstrating a remarkable strength. In modern architecture, in which building conventions underlie stringent economic constraints, framework and material-intensive domes have often been replaced by cost-efficient, but less performative grid-shells covered by pre-fabricated, planar elements [9,10]. Such grid-based constructions, in which building parameters, e.g. grid-size, determine structural stability, are restricted in their architectural degree of freedom [10]. These disadvantages can be countered by the development of segmented shell constructions, which combine the advantages of structural strength and freedom of design. Segmented shells thus represent economically optimized structures by using pre-fabricated elements [10]. The multi-plated echinoid skeleton can be used as a biological role model for developing constructional principles that can be transferred into biomimetic solutions for improved segmented designs [9].

### 1.1. The echinoid role model

The echinoid (Echinodermata: Echinoidea) skeleton is constructed of multiple biomineralized plates [11], which are securely interconnected by soft tissues and, in some cases, skeletal interlocks [12–17]. The hierarchical organization of echinoids additionally allows for the separation and functional interpretation of principles along the skeletal configuration [18]: the complete individual includes all skeletal elements, soft tissues and appendages (hierarchical level 1). The denuded echinoid test (hierarchical level 2), which encloses the internal organs, serves as a platform for appendages and is made of multiple plates (hierarchical level 3). Individual skeletal elements consist of a lattice-like stereom (hierarchical level 4) with a highly differentiated morphology, density and function [11,19,20]. The stereom is made up of trabeculae (hierarchical level 5) consisting of high-magnesium calcite struts [21–23], which represents a composite material (hierarchical level 6) with 0.1–0.2 wt% organic substances [24,25].

The architecture of the sea urchin skeleton has been in the focus of technical analysis for the last decades [9,14–16,26–30]. In-depth studies have been made on the test's microstructure using scanning electron microscopy (SEM) and increasingly micro-computed tomography (µCT) [11,19,20,31–38]. The most comprehensive technical studies on the echinoid skeleton provided for a detailed theoretical and mechanical background, which were discussed with respect to dome structures and their static properties [15,26,28]. The plate connections and internal supports were also described as structures that support the test under compression loads, whereas connective tissues counteract tension stress in most echinoids [15,16].

Clypeasteroid echinoids (Echinoidea: Clypeasteroidea) are usually flattened sea urchins featuring various internal support systems such as pillars, buttresses and ridges [9,15,16,31,38]. These echinoids can be abundant in high-energy environments and the fossil record, where they are frequently well preserved [39–42]. The occurrence of *Echinocyamus pusillus* under these conditions demonstrates the remarkable test strength, which is attributed to the presence of skeletal reinforcements [9,43,44].

**Figure 1.**
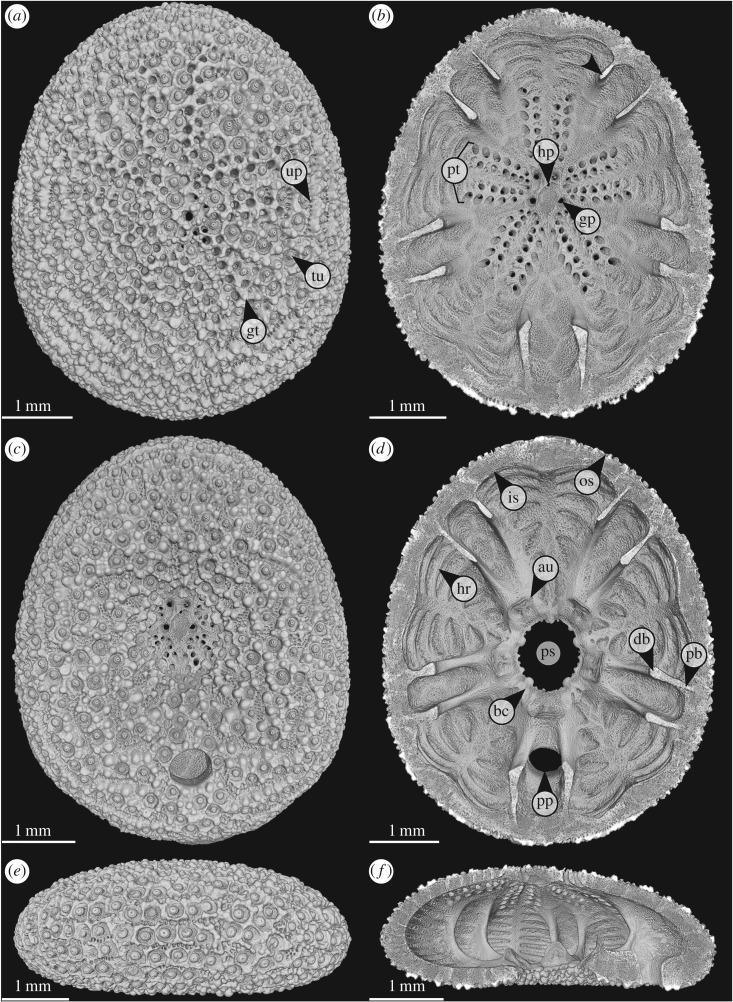
*Echinocyamus pusillus*. Micro-CT-based volume rendering of the denuded test [GPIT/EC/00740:gg-al-1.73]. (*a*) View of the aboral side. (*b*) Horizontal section with view onto the internal aboral surface. (*c*) View of the oral side. (*d*) Horizontal section with view onto the internal oral surface. (*e*) Lateral view, anterior to the left. (*f*) Lateral section showing internal test structures. Abbreviations: au, auricle; bc, basicoronal ring; bu, buttress; db, distal buttress; gp, genital pore; gt, glassy tubercle; hp, hydropore; hr, horizontal rib; is, inner surface; os, outer surface; pb, proximal buttress; ps, peristome; pt, petal; pp, periproct; tu,tubercle; up, unipore.

The clypeasteroid genus *Echinocyamus* is characterized by a minute test size (typically less than 20 mm in length) [45–50]. *Echinocyamus* is abundant in Recent and fossil environments distributed around Europe, West Africa, southeastern USA and from the Indo-Pacific, where it occurs from shallow waters to the deep sea [48,51–59]. The common species *E. pusillus* (figure 1) has been broadly described with regard to its morphology [45,54,59–61] and ecology [46,62–65]. One exceptional characteristic of this echinoid is that it lacks collagenous fibres within its sutures [15,16]. The stability of the test therefore relies primarily on the skeletal construction [16].

Although it has been shown that echinoids can, in general, be used as role models in engineering sciences [9,14–18,26,28,30,35], only little research has yet focused on the technical aspects of the unique *E. pusillus* test in particular [16]. Major aspects responsible for the test stability such as the three-dimensional interaction of test structures and variations in the stereom density remained hitherto unaddressed. The skeletal features that lead to this remarkable test strength are explored here in detail using X-ray µCT and SEM. The use of three-dimensional techniques combined with high-resolution SEM imagery allows for in-depth analyses of structures and their interactions for a novel functional interpretation. The understanding of the strengthening mechanisms is not only important as the basis for technical analyses and potential biomimetic applications, but also a contribution to palaeontology where these echinoids are used to reconstruct ancient ecosystems and biotic interactions [43,46,47,50,66].

### 1.2. Load model

**Figure 2.**
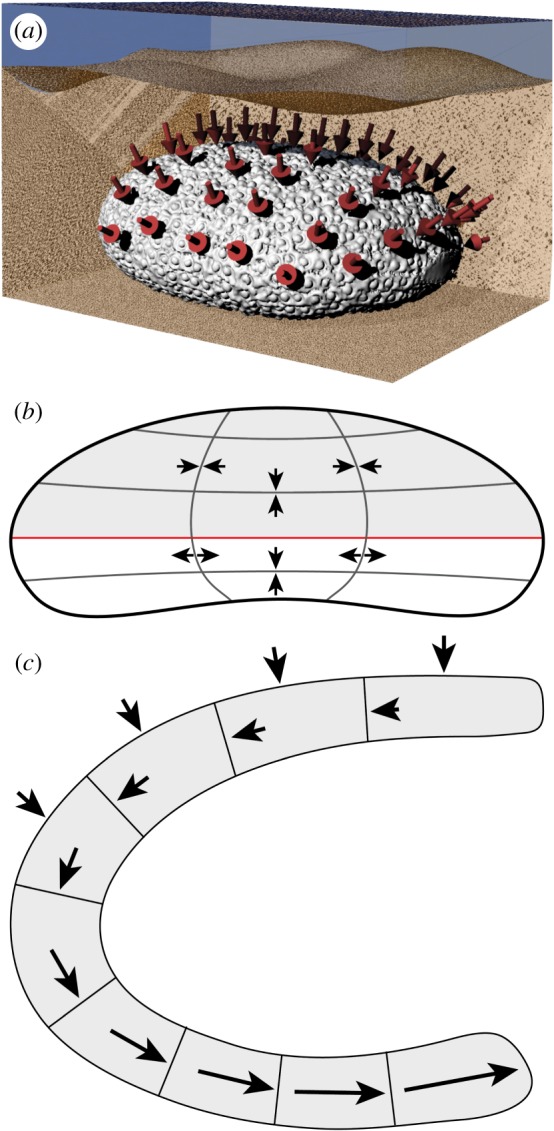
Load conditions in *E. pusillus*. (*a*) Test rendered in a hypothetical environment, buried within the sediment. Arrows indicate the loaded areas [GPIT/EC/00740:gg-al-1.73]. (*b*) Sketch of *E. pusillus* in lateral view. Radial lines indicate the course of radial stress. Horizontal lines represent the course of latitudinal stress. The red line indicates the ambitus. (*c*) Sketch of a part of the test in lateral view. External arrows indicate downward stress that turns into lateral thrust within the plates.

*Echinocyamus pusillus* is known to live infaunally and burrows into various substrates ranging from silts to coarse sands [53,63–65,67,68]. The skeleton is covered by a thin sediment layer [64], which causes downward loads on the test. The roughly dome-shaped test is considered to behave as a three-dimensional array of arches [15], on which downward forces are turned into lateral thrust [69] (figure 2). The flattening of these arches causes additional thrust in the test [15]. The load model used here is simplified, disregarding possible lateral loads caused by water agitation and the respective sediment movement. Water pressure has been shown to have no effect on the echinoid skeleton, as the pressure within the skeleton is similar to that of the outside [70].

Finite-element analyses based on slightly oblate regular sea urchin skeletons revealed that circumferential forces are compressive along the entire tests, increasing from the area of origin to the oral side (bottom) of the test [26,28] (figure 2). The radial forces result in compressive stress at the area of origin and along horizontal elastic trusses, whereas radial forces in all other areas result in tensile stress [26]. The structural layout of *E. pusillus* is analysed and interpreted with respect to the preceding load model.

## 2. Material and methods

### 2.1. Materials

Denuded tests of *E. pusillus* were collected in the summer of 2010 during SCUBA dives around the island of Giglio (Tyrrhenian Sea, Italy: 42°21′07.9^″^ N 10°52′52.1^″^ E) and from beach sediments at Riccione (Adriatic Sea, Italy: 44°01′17.9^″^ N 12°38′13.6^″^ E) in September 2014. The samples are stored at the Department of Geosciences, University of Tübingen, Germany, under repository GPIT/EC/00740 for Giglio and GPIT/EC/00741 for Riccione. GPS data are obtained from Google Maps 2017.

### 2.2. Methods

The test of *E. pusillus* is analysed using µCT and SEM with respect to the: (i) test design, including radial curvature, plate arrangement and thickness, (ii) plate architecture, including stereom types and stereom density, (iii) interlocking mechanisms on different hierarchical levels, and (iv) internal support system with respect to stereom differentiation and sutural layout.

#### 2.2.1. Micro-computed tomography

Specimen GPIT/EC/00740:gg-al-1.73 was cleaned for 30** **min in a Bandelin DT106 (Bandelin Electronic, Berlin, Germany) ultrasonic bath and then air-dried. The scan [71] was generated with a Phoenix Nanotom 180 nF (General Electric Company Corporation, Boston, MA, USA) at the German Aerospace Center (Deutsches Zentrum für Luft- und Raumfahrt), Stuttgart, Germany. The isotropic voxel resolution is 3** **µm (voltage = 80** **kV, power = 180 µA, exposure time = 800** **ms, projections = 2000).

#### 2.2.2. Scanning electron microscopy

Complete tests of *E. pusillus* [GPIT/EC/00741] were placed on separate aluminium specimen stubs (Plano GmbH, Wetzlar, Germany) in either the horizontal (oral side down) or lateral (right side down) position. Tests were attached to the stubs by partially impressing the echinoid skeleton into hand-warm Leit-C-Plast (Plano GmbH, Wetzlar, Germany) on a Leit-Tabs (Plano GmbH, Wetzlar, Germany) base. The non-Leit-C-Plast embedded part of the skeleton was then ground in the horizontal or lateral plane employing a Dremel 300i rotary tool (Dremel, Racine, WI, USA) equipped with a Dremel EZ Speedclic diamond cutting wheel (Dremel, Racine, WI, USA). The ground surface was additionally fine-sanded with 1200-grit sandpaper. Grinding residues were removed by compressed air. Samples GPIT/EC/00741:RI-n.5, RI-1.6 and RI-1.19 were additionally placed for 1** **s in hydrochloric acid (2.8** **mol l^−1^) to expose the interlocking mechanism more clearly. Acid-treated samples were washed three times in water to stop the reaction and removing the acid. Samples were then cleaned in a 30** **min Bandelin DT106 (Bandelin Electronic, Berlin, Germany) ultrasonic bath. Samples were dried in a Heraeus LUT6050 (Kendro Laboratory Products, Hanau, Germany) convection oven at 60**°**C and then platinum-sputtered on a planetary rotation disc for 120** **s using a Baltek SCD 005 sputter coater (Bal-Tec, Balzers, Lichtenstein). Samples were scanned with a Leo 1450VP (Carl Zeiss AG, Oberkochen, Germany) scanning electron microscope at the Department of Geosciences, University of Tübingen, in secondary electron (SE1) mode at 9 or 15** **kV. Micrographs are recorded to a resolution of 2048 × 1536 pixels per inch (ppi) for standard images and 3072 × 2304 ppi for a high-resolution overview [GPIT/EC/00741:RI-1.19].

#### 2.2.3. Test design

The µCT scan was rendered in Avizo 9.2.0 (Thermo Fisher Scientific, Waltham, MA, USA). The radial curvature was determined from µCT sections through the centre of the test. In these sections, the centre of each plate was identified. When the connecting line between the plate centre points matches a monotonic curve progression along the entire section, the test is considered convex. The plate arrangement is described from horizontal µCT sections visualized in Avizo. The sections are examined for the sutural course throughout the test in three-dimensional space. The plate thicknesses are determined from horizontal µCT sections and measured orthogonal to the test surface. The measurements were obtained from three sections and for all 20 plate columns, resulting in 3 × 10 data points from ambulacral plates and, respectively, 3 × 10 data points from interambulacral plates. The sutures were measured at the boundaries between ambulacral plates (perradial suture), between interambulacral plates (interradial sutures) and between ambulacral and interambulacral plates (adradial sutures). Perradial and interradial sutures occur five times on each of the three slices (3 × 5 data points), whereas adradial sutures occur 10 times on each of the three slices (3 × 10 data points).

The thickness measurements are statistically tested for differences among the corresponding regions using a Kruskal–Wallis *H* test followed by a pairwise Benjamini, Hochberg and Yekutieli *p*-adjusted Wilcoxon post hoc analysis [72]. Statistical analyses are computed using the R software environment (R Foundation, Vienna, Austria) v. 3.3.2 [73].

#### 2.2.4. Plate architecture

The main stereom types found within the plates are assigned to established stereom types [19]. The plates' outer surfaces are excluded from this analysis owing to the complex microstructures involving numerous stereom types [19] that do not contribute to the overall test stability. Stereom densities are analysed using three subvolumes with an edge length of 30 voxels (90** **µm) along transects from the centre to the plate boundaries (electronic supplementary material). Three transects per plate (three plates in total) at an angle of roughly 120° to one another are used.

The subvolumes are processed and analysed in Avizo 9.2.0. The delineate sharpening filter (size = 3 pixels) of Avizo was used to enhance the contrast between air and the stereom within each raw subvolume. The denoised subvolumes were then binarized applying the build-in auto-thresholding function on each three-dimensional subvolume (mode = min–max, criterion = factorization). This automated method of thresholding ensures repeatability of the procedure and eliminates experimenter bias. The built-in analyse function reports the amount of positive (stereom) and negative (non-material) voxels for each three-dimensional volume. Stereom density is the sum of positive voxels divided by the total number of voxels within the subvolume.

Stereom density distributions are statistically tested for differences among the three plate regions using a Kruskal–Wallis *H* test followed by a pairwise Benjamini, Hochberg and Yekutieli *p*-adjusted Wilcoxon post hoc analysis [72]. Statistical analyses are computed using the R software environment [73].

#### 2.2.5. Interlocking mechanisms

Interlocking mechanisms are analysed from two hierarchical levels: the plates and the stereom. The plates are examined with respect to the three-dimensional morphology and the resulting interaction with neighbouring plates along the sutures. Sutures were observed from translations throughout the µCT section. A suture is recognized as sinuous, when the plate's margins are curved, thus allowing for interlocking with neighbouring plates. The plate's microarchitecture is examined for the stereom types involved in the plate [19]. The segmentation of a single ambulacral plate from the oral side was performed in Avizo in manual mode, using the brush-tool with masking enabled (masking range: 82–255). The surface was rendered and smoothed (unconstrained smoothening, smoothening extent = 9). The surface was additionally smoothed using the smooth surface command (iterations = 10, *λ* = 0.9).

The interaction between plates on the microstructural stereom level is analysed for interlocking mechanisms based on SEM images. The mechanism of interlocking between plates is divided into three categories: (i) rod-like trabecular protrusions penetrate deeply into the stereom interspace of adjoining plates, (ii) knob-like trabecular protrusions interlock with depressions formed by the opposing plate's stereom without penetration into the stereom interspace, and (iii) the trabecular protrusions of one plate interlock with trabeculae of the neighbouring plates developing a form-closed interlocking hook.

#### 2.2.6. Internal support system

The buttress system is analysed for its overall morphology, including its course, length and plate involvement, as well as for its sutural layout, and variations in stereom density. Based on µCT and SEM data, the origin of the buttresses' plates is examined together with its course throughout the test. Length measurements are based on a horizontal µCT section. The sutural layout is examined for its position and interlocking mechanisms. The stereom density of the buttresses is measured at three intervals between the proximal end of one buttress with a subvolume edge length of 20 voxels (60** **µm). The measurements and volumetric and statistical analyses follow the protocols under **‘**Plate architecture’.

#### 2.2.7. Figure processing

Images are adjusted for brightness, contrast and colour using Photoshop CC 2017 (Adobe Systems, San Jose, CA, USA). Figure 2*a* was rendered using the three-dimensional modelling software Rhinoceros 5 (McNeel, Seattle, WA, USA) with a surface model of the echinoid's tests based on µCT scan processed in Avizo. Line drawings are generated in Adobe Illustrator CC 2017 (Adobe Systems, San Jose, CA, USA). Zerene Stacker 1.04 (Zerene Systems, Richland, WA, USA) is used to stack figure 4*a* (mode: DMAP, rendering: Lanczos8 16 × 16) from four µCT sections. Figure 8*a* is stitched from two SEM micrographs using Autostitch (University of British Columbia, Vancouver, BC, Canada). Figures were processed using Adobe InDesign CC 2017 (Adobe Systems, San Jose, CA, USA).

## 3. Results

### 3.1. Test design

The test of *E. pusillus* is sub-elliptical in shape in top view (figure 1*a*). In radial section, the test is flattened and convexly shaped without concave elements (figure 1*f*). The test's radial curvature is convex over the entire surface, including the region around the slightly infundibulate peristome. The nearly dome-shaped form of the test represents a double-curved shell construction. The test's outer surface shows numerous microscopic surface features, including tubercles, glassy tubercles, genital and ocular pores, single hydropore, ambulacral pores, unipores, the centrally located peristome and the periproct that lies between the two posterior buttresses on the oral surface (figure 1). The paired ambulacral pores of the petals and serially arranged unipores lie flush with the outer test surface. The basicoronal ring consists of five fused basicoronal interambulacral plates and five pairs of narrow basicoronal ambulacral plates (figure 1*d*).

**Figure 3.**
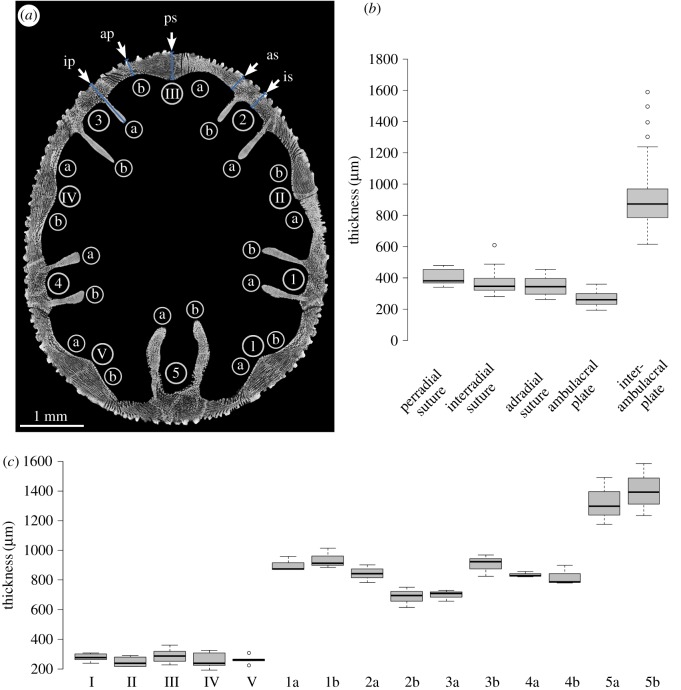
Analyses of test thickness. (*a*) Horizontal µCT section of *E. pusillus* indicating measurement points for plate thickness. Terminologies following Lovén [74] [GPIT/EC/00740:gg-al-1.73]. (*b*) Boxplot diagram comparing thicknesses of perradial, adradial and interradial sutures with the thickness of ambulacral and interambulacral plates. (*c*) Boxplot diagram showing the thicknesses of plates. Ambulacral plates a and b are pooled to a single value (I – V). Interambulacral buttress length is given for each plate individually for plates a and b (1–5). Plate nomenclature following Lovén [74].

The test of *E. pusillus* varies in thickness (figure 3). The ambulacral plates are on average 260.74 ± 47.9** **µm (*n* = 30) thick and thinner than the elongated interambulacral plates (buttresses) with an average thickness of 872.48 ± 132.3** **mm (*n* = 30) (figure 3 and table 1). The plates of the anterior ambulacra (II, III and IV) are on average thinner than those of the posterior ambulacra (I and V) (table 1). The perradial suture between ambulacral plates measures on average 380.94 ± 41.0** **µm (*n* = 15) in thickness. The interradial sutures between interambulacral plates show an average thickness of 346.25 ± 60.4** **µm (*n* = 15). Adradial sutures between ambulacral and interambulacral plates are 343.50 ± 72.3** **µm (*n* = 15) thick on average. A Wilcoxon test shows that suture areas are thicker than the centre of the ambulacral plates (*W* = 1670, *p* < 0.001, *n* = 90). A pairwise Benjamini, Hochberg and Yekutieli *p*-adjusted Wilcoxon post hoc analysis indicates that the average plate thickness at the perradial and interradial sutures does not show statistical differences (*p* = 0.103, *n* = 30). This also applies to the comparison between perradial and interradial sutures (*p* = 0.433, *n* = 30), and between adradial and interradial sutures (*p* = 1.000, *n* = 30).

**Table 1.** Sutural thickness and stereom density measurements of the test of *E. pusillus*. mad, median absolute deviation; *n*, sample size.

		median	mad	minimum	maximum	*n*
plate thickness (µm)	ambulacral	260.74	47.94	193.10	359.83	30
	buttress	872.48	132.26	614.91	1586.68	30
suture thickness (µm)	perradial	380.94	40.96	340.52	479.65	15
	interradial	346.25	60.36	280.49	607.03	15
	adradial	343.50	72.28	262.14	454.35	15
stereom density (%)	suture	49.43	3.20	47.27	54.12	9
	galleried stereom	45.35	2.92	40.77	49.96	9
	labyrinthic stereom	48.01	1.49	44.74	50.43	9
	total	48.01	2.89	40.77	54.12	27

### 3.2. Plate architecture

**Figure 4.**
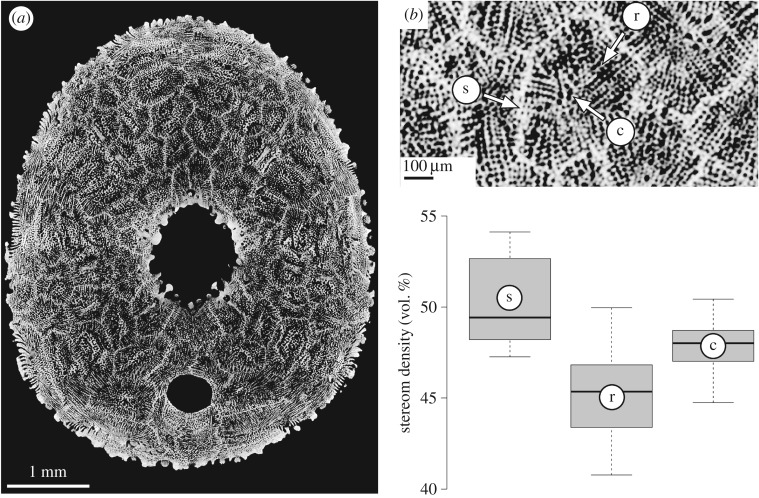
Stereom differentiation in *E. pusillus* [GPIT/EC/00740:gg-al-1.73]. (*a*) Micro-CT section of the oral side showing the mosaic of plates. (*b*) Close-up indicating three analysed regions of the plate: *c*, unordered labyrinthic stereom at the plate's centre; *r*, directional galleried stereom at the plate's rim; *s*, directional galleried stereom within sutures. (*c*) Comparison between three prominent areas of the plates.

The skeletal plates are subdivided into two clearly differentiated regions: the (i) plate's centre consisting of the unordered labyrinthic stereom, and the (ii) surrounding distal plate areas consisting of galleried stereom (figure 4). The latter is constructed by highly ordered parallel struts, which run perpendicular to the plate sutures. The galleried stereom at the sutures is of higher stereom density than that of the remaining plate, as indicated by the brighter grey level in the µCT section (figure 4).

The analysed ambulacral plates show an average stereom density of 48.0 vol.% (*n* = 27) (figure 4 and table 1). Stereom densities vary between the labyrinthic stereom at the plates' centre (48.0 vol.%, *n* = 9), the galleried stereom at the centre surrounding areas of the plates (45.3 vol.%, *n* = 9) and the galleried stereom at the sutures (49.4 vol.%, *n* = 9). A Kruskal–Wallis *H* test followed by a Benjamini, Hochberg and Yekutieli *p*-adjusted Wilcoxon pairwise comparison indicates that the stereom densities differ between the galleried stereom at the plate sutures and the galleried stereom at the plate distal area (*p* = 0.015, *n* = 27). The average density of the galleried stereom at the suture is different than that on the plate's centre. This difference is, however, not significant (*p* = 0.127, *n* = 27). The density of the galleried stereom at the plate's suture likewise differs from that on the plate's centre, though not significantly (*p* = 0.127, *n* = 27).

### 3.3. Interlocking mechanisms

**Figure 5.**
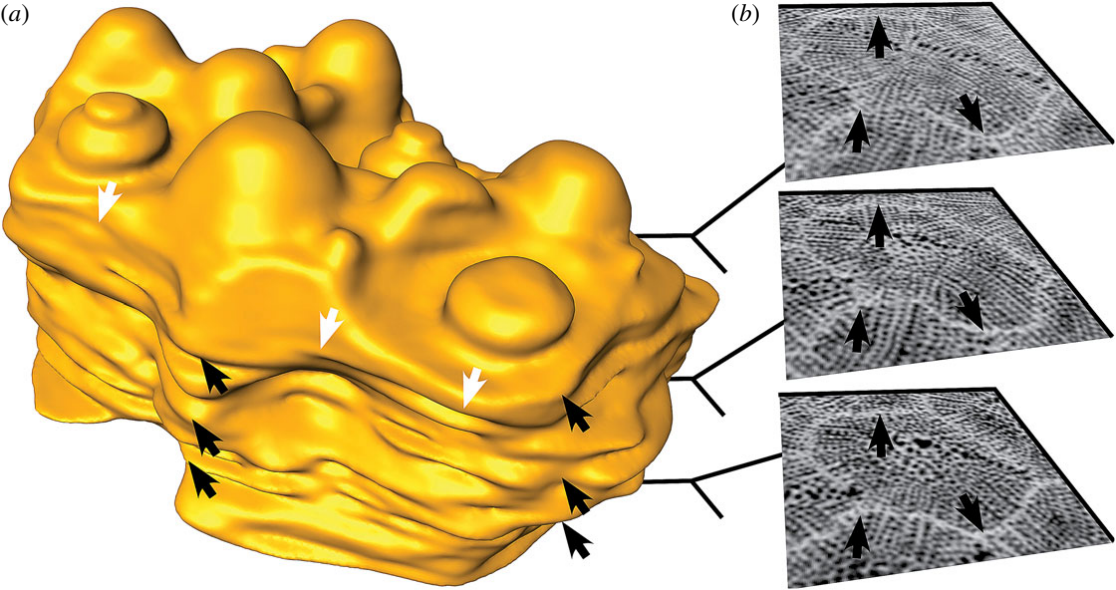
Plate shape of *E. pusillus* [GPIT/EC/00740:gg-al-1.73]. (*a*) Three-dimensional surface rendering of a single segmented plate. Sutures follow a sinuous path in all directions. White arrows indicate the sinuous course in horizontal plane, black arrows in lateral direction. (*b*) Micro-CT sections of the segmented plate showing the different plate outlines at three intervals. Arrows correspond to black arrows in (*a*).

**Figure 6.**
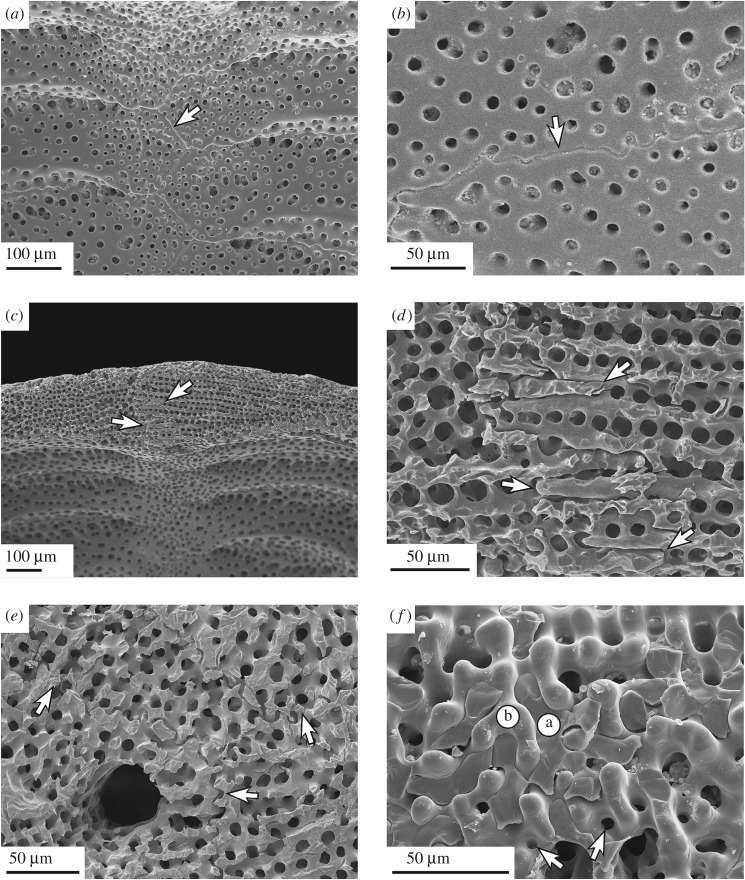
SEM micrographs of plate joints in *E. pusillus*. (*a*) Sinuous course of sutures within a perradial boundary [GPIT/EC/00741:RI-n.4]. (*b*) Sutural interlocking in detail. Knobs reaching from one into another plate can increase test strength [GPIT/EC/00741:RI-n.4]. (*c*) Trabecular interlocking at a thickened perradial suture [GPIT/EC/00741:RI-n.5]. (*d*) Trabecular interlocking in detail, where trabeculae from one plate protrude into the stereom interspace of an adjoining plate [GPIT/EC/00741:RI-n.5]. (*e*) Sutural interlocking in a three-dimensional mosaic of surrounding plates. Arrows indicate plate boundaries [GPIT/EC/00741:RI-1.6]. (*f*) Orthogonal view onto a suture. Fractured trabeculae are attached and penetrate into the interspace of another plate; a and b indicate the affiliation of the involved plates. Arrows indicate narrow depressions for knob-like interlocking [GPIT/EC/00741:RI-n.4].

Plate interlocking occurs at two hierarchical levels. The irregular outline of the plates (figure 5) is integrated into a complex mosaic of plates (figure 4). Sutural interlocking can be seen on the inner surface of the test, where plate edges feature a high degree of sinuosity, leading to an interdigitation of plates (figure 6). The outer surface of the test shows intense intergrowth where individual plates and plate boundaries are hardly visible, because they are masked by surface microstructures, such as tubercles and glassy tubercles (figure 1). Trabecular interlocking occurs where trabeculae of the galleried stereom meet the suture in a perpendicular direction (figure 6). These interlockings can be present as interdigitating rod-like structures, where trabeculae penetrate deeply into the opposing stereom interspaces, or knob-like structures, that interlock with the adjoining plate's surfaces. Rod- and knob-like interlockings are distributed over the entire area of the sutures. Fusion between single trabeculae of adjoining plates can be present, but is rare.

### 3.4. Internal support system

**Figure 7.**
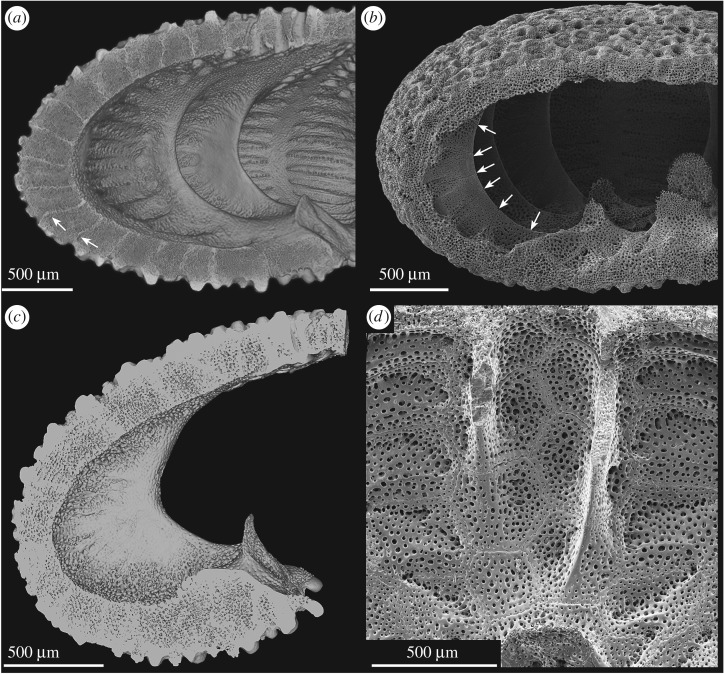
Internal buttress system in *E. pusillus*. (*a*) Lateral section of a µCT-based volume rendering showing the internal supports emerging from fused auricles. Arrows indicate sinuous course of plate boundaries [GPIT/EC/00740:gg-al-1.73]. (*b*) SEM micrograph of segmented buttresses. Arrows indicate plate sutures [GPIT/EC/00741:RI-1.19]. (*c*) Micro-CT section of a single buttress showing its widest extent at the ambitus. (*d*) SEM micrograph of two internal buttresses showing the supports as protrusions of interambulacral plates [GPIT/EC/00741:RI-1.2].

**Figure 8.**
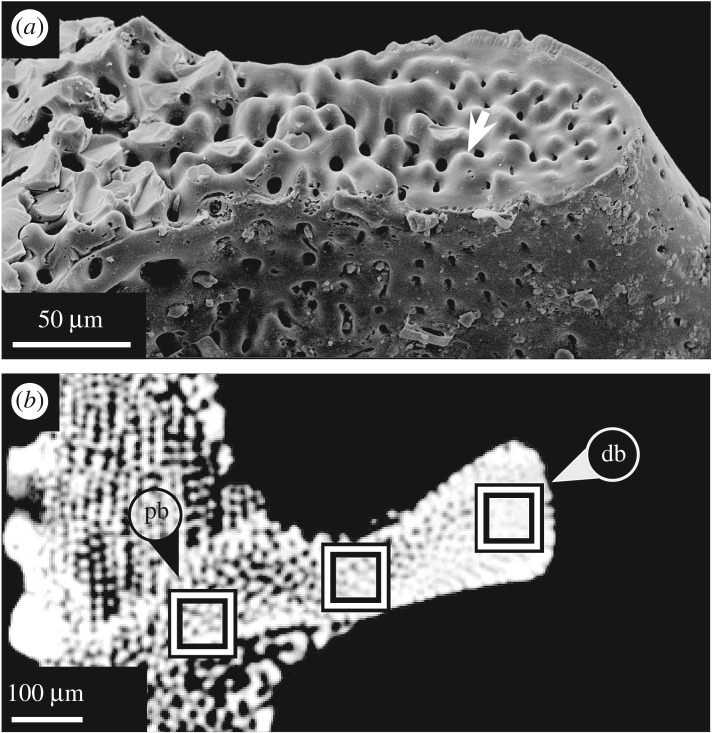
Horizontal section of buttresses in *E. pusillus*. (*a*) SEM micrograph of a buttress suture. Individual buttress segments are interconnected by knob-like structures [GPIT/EC/00741:RI-1.29]. (*b*) Micro-CT section indicating the stereom density distribution within a buttress. Location of rectangles indicates areas used for stereom density comparison [GPIT/EC/00740:gg-al-1.73]. db, distal buttress; pb, proximal buttress.

The internal support system in *E. pusillus* consists of two structural elements: the radial buttress system and longitudinal ribs (figure 1*d*,*f*). The buttress system is exclusively constructed from elongated interambulacral plates emerging from the outer limits of the petals and extending towards the fused auricles at the basicoronal ring. Buttresses are segmented by horizontal or slightly inclined sutures (figure 7). These sutures are interconnected by rod-like and knob-like trabecular protrusions at the proximal regions, and exclusively by knob-like trabecular protrusions at the distal regions (figure 8). The buttresses are longest at the ambitus. In horizontal section, individual buttresses are club-shaped and merge with the interambulacral plates in a V-shape (figure 7). Buttresses vary in length between 614.91 and 1586.68** **µm (table 1). The posterior buttresses 5a and 5b (figure 3) that enclose the periproct are longest (table 2). Buttresses 2b and 3a are shorter than 2a and 3b, and buttresses 1b and 4a are longer than 1a and 4b.

**Table 2:** Plate thickness (µm) measurements of the test of *E. pusillus*. mad, median absolute deviation, *n*, sample size. Roman numerals indicate ambulacral plates; arabic numerals indicate interambulacral plates. Small letters indicate the position of the plate according to Lovén [74].

	median	mad	minimum	maximum	*n*
Ia	277.89	42.02	236.87	306.23	30
Ib	275.64	22.25	260.63	299.24	30
IIa	258.55	44.64	216.20	288.66	30
IIb	219.29	5.16	215.81	278.14	30
IIIa	260.85	16.92	249.44	319.52	30
IIIb	311.87	71.11	225.34	359.83	30
IVa	246.34	35.82	222.18	324.03	30
IVb	231.47	56.89	193.10	306.19	30
Va	264.62	15.17	254.39	305.59	30
Vb	257.12	11.82	223.36	265.09	30
1a	874.99	7.46	869.96	958.87	30
1b	912.49	37.75	887.03	1015.76	30
2a	842.63	85.43	785.01	904.77	30
2b	697.39	80.83	614.91	751.91	30
3a	710.37	30.22	657.91	730.75	30
3b	924.85	64.83	826.74	968.58	30
4a	830.73	12.69	822.17	857.39	30
4b	787.20	8.42	781.52	899.82	30
5a	1299.45	182.06	1176.65	1493.49	30
5b	1392.38	230.08	1237.19	1586.68	30

Whereas buttresses are extensions of the interambulacral plates, longitudinal ribs are extensions of the ambulacral plates running perpendicular to the internal buttress systems (figure 7). These ribs extend from the buttresses into the thickened and planar perradial sutures (figure 1). The ribs are most prominent at the oral side, becoming gradually less protuberant towards the petalodium. The plates and ribs are offset, causing a discontinuous suture in the horizontal direction. The stereom density of the buttress varies along its length (figure 8). The distal region is of highest density (92.0% stereom), the centre is of lowest density (68.3% stereom) and the proximal end is of intermediate density (83.3% stereom).

## 4. Discussion

### 4.1. Test design

The overall radial test curvature of *E. pusillus* is convex (figure 1). This shape is not interrupted by any indentation throughout the test. The radially convex curvature allows for transmission of radial forces along the plates without changes in their direction and thus contributes to a stable test construction. The test of *E. pusillus* features numerous apertures, including the prominent peristome and periproct as well as ambulacral pores, unipores, genital pores, ocular pores and the single hydropore. The unipores are abundant at the longitudinal sutures and can be considered as potential weak points in the test [16]. The peristome is a large aperture in the echinoid skeleton [26], which needs reinforcement. In *E. pusillus*, this is achieved by the fused auricles of the basicoronal ring, which thicken the peristome's margin (figure 1*d*). This structural reinforcement distributes stress around the aperture [26]. Additionally, the buttress system merges into the fused auricles (figure 1*d*). This construction not only reinforces the basicoronal ring by further thickening, but also represents a T-beam construction that provides strength against bending [75].

The echinoid skeleton typically represents a light-weight structure [18]. The overall test density and thus the amount of structural material in the test of *E. pusillus* are less than 50%. Those areas where higher stresses are expected to occur, such as within the buttress system, show corresponding higher stereom densities. By contrast, lower densities are present where less stress is expected to occur such as in the centre of plates and buttresses.

### 4.2. Plate architecture

The plates of *E. pusillus* incorporate two stereom types, a centrally located region of unordered labyrinthic stereom and a surrounding area of strictly ordered galleried stereom (figure 4*b*). The labyrinthic stereom can distribute stress equally in multiple directions of the plate's plane [76], which reduces the accumulation of stress. The galleried stereom is able to transfer load forces directionally along its longitudinal struts, which lie perpendicular to the sutures. The combination of these two stereom types is an important structural feature of the plates. Applied downward loads are firstly transferred into lateral thrust. This thrust is then distributed equally along the roughly dome-shaped shell, which reduces stress accumulation in a specific area. The galleried stereom can transfer stress to a neighbouring plate where it, again, is further distributed onto surrounding plates.

### 4.3. Interlocking mechanisms

In segmented shell constructions, plate joints are usually considered as weak points, where stress accumulation can lead to structural failure [30]. The plate joints in *E. pusillus* show three strengthening mechanisms: (i) sutural interlocking based on the irregular and sinuous course of plate boundaries at the hierarchical level of individual plates, (ii) trabecular interlocking based on rod-like, knob-like or hook-like trabecular protrusions at the hierarchical level of the stereom (figure 6), and (iii) thickening of the contact areas between adjoining plates (figure 3).

Sutural interlocking relies on the shape of the entire plate. The irregular boundary regions between plates lead to a three-dimensional interlocking along the mosaic of plates (figure 5). Such a three-dimensional interlocking reduces the degree of freedom for plate movement. Trabecular interlocking is present on a lower hierarchical level where trabeculae of one plate reach into the stereom interspace of another (figure 6). Knob-like interlocking are found when the abutting stereom interspace is too narrow for the trabeculae to penetrate (figure 6*f*). At the internal surface of the test, knobs, hooks and occasionally fusion of single trabeculae between neighbouring plates can increase the strength of plate cohesion (figure 6) [16,49]. Sutures at the inner surface are predominantly present as clear plate boundaries, whereas sutures on the outer surface are often tightly connected to one another, resulting in a homogeneous surface.

Sutural thickening increases the area of plate connections. An increase in the contact area of adjoining plates can thus boost the performance of both the sutural and the trabecular interlocking mechanisms by providing a larger contact area. The thickened sutures act as additional internal buttresses, enhancing stress transmission between the oral and aboral side [16]. The sutural design in *E. pusillus* is considered to have a high significance for the shell strength. These three strengthening mechanisms lead to extensive plate connections with tight-fitting sutures. These interlocking mechanisms result in a segmented construction that behaves as a monolithic structure, characterized by a load transfer with reduced stress accumulation on the sutures. The monolithic behaviour, combined with the advantages of the two incorporated stereom types, results in a segmented shell construction with beneficial load transfer performance.

### 4.4. Internal support system

The internal support system is one of the most prominent reinforcement structures of the test and contains the buttresses as well as longitudinal ribs [16,76] (figure 1). The constructional design conjoins the top and bottom of the test with buttresses sharing the loads. The presence of buttresses therefore reduces tensile stress at the lower parts (figure 2), allowing for test flattening [16,76]. The buttress arrangement follows the bilateral symmetry of the test (figure 3). The fact that one buttress of a corresponding pair is shorter than the other has an ontogenetic origin as in juvenile clypeasteroids; the interambulacral plates are inserted into the double-plate column one by one [61], becoming an a or b plate (figure 3*a*).

The multi-plated buttress system is formed from elongated interambulacral plates with sutures perpendicular to the surface (figure 7*b*). These sutures are inclined to the test's horizontal plane such that lateral thrust, which follows the arched ring, meets the sutures in a perpendicular direction (figure 2*c*). Sutures, onto which stress impinges perpendicularly, is subject to less shear stress [77]. In addition, plates of the buttresses are interconnected by knob-like stereom protrusions which prevent horizontal movement between plates. The microstructure of the buttresses also reveals a load-carrying design; the outer regions of the buttresses located towards the centre of the test show a higher density than the buttresses' limbs (figure 8). The structurally reinforced distal areas of the buttress system can improve the load transfer while maintaining structural integrity.

## 5. Conclusion

(i) The skeleton of *E. pusillus* is subject to various load conditions. Owing to the buried, infaunal mode of live, the weight of the sediment represents a continuous force acting on the skeleton.
(ii) The skeleton is a light-weight construction with an average stereom density of less than 50%. Despite this fact, the test shows a high preservation potential in the fossil record, indicating a remarkable strength.
(iii) Skeletal strength is achieved by multiple reinforcements along the hierarchical levels, including (a) the arrangement of plates in a mosaic pattern, (b) stereom differentiation within a plate which responds to different stress, (c) sutural interlocking due to an irregular plate outline, (d) various trabecular interlockings of neighbouring plates, (e) sutural thickening boosts area of interlocking mechanisms, and (f) internal support system that adds to the resilience of the test.
(iv) Plate interlocking leads to a construction that behaves as a monolithic structure. Together with the functional differentiation of the stereom within the plates and the double-curved form, the test exhibits an advantageous load-bearing performance.
(v) The skeletal adaptations of *E. pusillus* contain numerous reinforcing structures that can be of high interest as a role model in civil engineering. An in-depth analysis of the test strengthening structures and the transfer into technical applications can enhance the development of biologically inspired segmented light-weight constructions.

## Supplementary Material

Data origin

## Supplementary Material

Plate measurements

## Supplementary Material

volumetric analyses

## Data accessibility

The unprocessed µCT data of *E. pusillus* are accessible via the Dryad Digital Repository under http://dx.doi.org/10.5061/dryad.rg54h [71].
